# Long-term atmospheric exposure to particulate matter and breast cancer risk: findings from a nested case-control study in France

**DOI:** 10.1038/s41416-025-03311-y

**Published:** 2026-01-13

**Authors:** Delphine Praud, Amina Amadou, Benoît Mercoeur, Margaux Duboeuf, Maryline Bouilly, Thomas Coudon, Lény Grassot, Elodie Faure, Florian Couvidat, Julien Caudeville, Bertrand Bessagnet, Pietro Salizzoni, Karen Leffondré, John Gulliver, Gianluca Severi, Francesca Romana Mancini, Béatrice Fervers

**Affiliations:** 1 Department of Prevention Cancer Environment, Centre Léon Bérard, Lyon, France; 2 Inserm U1052, Centre de Recherche en Cancérologie de Lyon, Lyon, France; 3 Inserm U1296 Radiations: Défense, Santé, Environnement, Lyon, France; 4 Centre de Recherche en Epidémiologie et Santé des Populations (CESP, Inserm U1018), Facultés de Médecine, Université Paris-Saclay, UPS UVSQ, Gustave Roussy, Villejuif, France; 5 National Institute for industrial Environment and Risks (INERIS), Verneuil-en-Halatte, France; 6 CITEPA, Technical Reference Center for Air Pollution and Climate Change, Paris, France; 7 Ecole Centrale de Lyon, INSA Lyon, Université Claude Bernard Lyon 1, Ecully, France; 8 University of Bordeaux, ISPED, INSERM, Bordeaux Population Health Research Center, UMR1219 Bordeaux, France; 9 Centre for Environmental Health and Sustainability, School of Geography, Geology and the Environment, University of Leicester, Leicester, UK; 10 Department of Statistics, Computer Science and Applications (DISIA), University of Florence, Florence, Italy

**Keywords:** Risk factors, Breast cancer, Cancer epidemiology

## Abstract

**Background:**

Airborne particulate matter (PM) is a complex mixture of particles thought to be associated with a range of adverse health effects, including female breast cancer. Current evidence on the association between PM and female breast cancer risk is inconsistent.

**Methods:**

This study investigated the association between long-term exposure to PM and breast cancer risk in a nested case-control study within the French E3N-Generation cohort including 5222 breast cancer cases identified over the 1990–2011 follow-up period and 5222 individually matched controls. Annual mean concentrations of PM_10_ and PM_2.5_ at participants’ residential addresses, were estimated using a land use regression model. Odds ratios (ORs) and 95% confidence intervals (CIs) were estimated using conditional logistic regression models.

**Results:**

ORs for each 10 µg/m^3^ increase in the average of PM_2.5_ and PM_10_ were 1.14 (95% CI: 0.99–1.30) and 1.08 (95% CI: 0.98–1.18), respectively. When restricted to invasive ductal and lobular carcinomas, ORs were 2.74 (95% CI: 1.05–7.15) for PM_2.5_ and 2.05 (95% CI: 1.11–3.78) for PM_10_. Comparable effects of PM exposure estimated by a chemistry transport model reinforces these findings.

**Conclusion:**

This study suggests a potential association between PM_2.5_ and PM_10_ exposure and breast cancer risk.

## Introduction

Female breast cancer is the most commonly diagnosed cancer worldwide and makes an important contribution to the global number of cancer deaths [1]. More than 2.3 million new cases and 685,000 deaths from female breast cancer were reported in 2020, and it is estimated that there will be over 3 million new cases and 1 million deaths annually by 2040 [1]. In 2022, there were 65,659 cases of female breast cancer and 14,739 related deaths in France, ranking it as the country with the highest incidence rate [1].

Lifestyle plays an important role in female breast cancer incidence [2–4]. However, epidemiological and laboratory findings suggest that some physical environmental factors such as air pollution may also be associated with a higher risk of female breast cancer which is in particular supported by large geographical variation in incidence [5–7].

Outdoor air pollution is a major public health concern related to a range of adverse health effects, including cancer, and was associated with an estimated 4.2 million premature deaths in 2019 [8]. Currently, the vast majority of the world population, particularly in urban areas, is exposed to particulate matter (PM) at levels above the World Health Organisation (WHO) health recommendation thresholds in their air quality guidelines [8]. Airborne PM is a complex mixture of components. These particles vary in size, shape and composition, and may include inorganic ions, metallic compounds, elemental carbon, organic chemical, and soil-related compounds. They are classified by their diameter. PM with a diameter of up to 10 µm (PM_10_) can be inhaled and cause adverse health effects. PM that are up to 2.5 µm in diameter (PM_2.5_) are classified as fine particles and can penetrate deep into the lungs, and even pass into the bloodstream [9]. The mean level of PM_2.5_ exposure may affect the histologic characteristics of healthy breast tissue, which may, in turn, contribute to the carcinogenesis of female breast cancer [10].

The biological plausibility of an association between PM_2.5_ and PM_10_ and female breast cancer is supported by potential mechanisms of action. PM_2.5_ and PM_10_ could promote oxidative stress which could induce cellular stress and thus DNA damage [11, 12]. In addition, certain components of PM_2.5_ and PM_10_, such as polycyclic aromatic hydrocarbons (PAHs), could bind to DNA to form PAH-DNA adducts, which promote the development and progression of tumour cells [13]. Finally, some of the substances aggregated in PM_2.5_ and PM_10_ e.g., nickel, which is one of the heavy metals, is thought to disrupt oestrogen activity by binding to oestrogen receptors [14, 15]. Since 80% of female breast cancers are hormone-dependent, this could play a role in carcinogenesis [16].

Evidence for an association between PM_2.5_ and PM_10_ and the risk of female breast cancer is inconsistent. A recent meta-analysis, based on 32 studies on PM_2.5_ and 27 on PM_10_ from cohorts in North America, Europe, and Asia, reported no clear association between long-term exposure to PM_2.5_ or PM_10_ and the incidence of female breast cancer [17]. Among the studies referenced in this meta-analysis [18, 19] and in more recent publications not included in the review [20, 21], several have reported positive associations between long-term exposure to PM_2.5_ and female breast cancer risk [18–21]. For instance, a population-based study in Denmark led between 2000 and 2014 reported an odds ratio (OR) of 1.21 (95% confidence interval (CI): 1.11–1.33) for breast cancer incidence per 10 µg/m^3^ increase in PM_2.5_ among all women and 1.32 (95% CI: 1.09–1.60) for women under 55 years old [21]. Another study in the UK found an association between PM_10_ levels in 2007 and cumulative mean annual average PM_10_ levels, and postmenopausal breast cancer risk [22]. The discordant conclusions between the studies could be explained by differences in the PM exposure assessment methods used, in terms of spatial resolution (from <100 metres to 10 kilometres), geographical coverage (regional to national coverage) and temporal resolution (data updated daily to data updated annually over a highly variable period which can extend over >10 years), as well as the variations in mean PM exposure in the study populations [17, 23]. In addition, some studies generally used addresses at inclusion or diagnosis as an approximation throughout the follow-up and some studies estimated exposure only at inclusion [17]. Given the long latency period of carcinogenesis, one of the main challenges is obtaining long-term exposure data to PM_2.5_ and PM_10_ that is spatially and temporally resolved at high resolution, at the individual residential level, using validated exposure models.

Here, we report results from a large French nested case-control study, investigating the association of long-term exposure to PM_2.5_ and PM_10_, estimated at the participants’ residential addresses over time, with female breast cancer risk.

## Materials and methods

### The E3N-Generation cohort

E3N-Generation cohort is an national, ongoing, prospective cohort study [24]. Between June 1990 and November 1991, 98,995 women born between 1925 and 1950 and insured by the French national health insurance scheme for national education workers (MGEN) who were living in continental mainland France were enrolled, after having given written informed consent (www.e3n.fr). This cohort is part of the European Prospective Investigation into Cancer and Nutrition (EPIC study), which included 10 European countries [25]. The objective of the EPIC study is to investigate risk factors for cancer and other chronic diseases in women. At inclusion, women completed a self-questionnaire about lifestyle, reproductive factors, anthropometry, medical history, including benign breast disease and gynaecological screening and familial history of cancer. After the initial inclusion in 1990, follow-up questionnaires were sent every 2 to 3 years thereafter. To date, 13 questionnaires have been sent with a mean participation rate of about 83%. Each follow-up questionnaire collected information on the occurrence of breast or other cancers and the reasons for any hospitalization or medical care at home, specifying the month and year of each event. A copy of the pathology report or any medical examination confirming the diagnosis of breast cancer was requested from the patient or the physician. Tumour characteristics, including histological type and hormone receptor status, were extracted from these reports [26]. Here, only data collected from the questionnaires up to 2011 have been analysed [24], and a rural or urban residential status at birthplace was assigned based on data from the most recent national census [27].

### XENAIR: a case-control study nested within the E3N-Generation cohort

XENAIR, a case-control study nested in the E3N-Generation cohort, has been previously described [28]. Breast cancer cases were identified by self-administrated questionnaires, insurance data or for around 1% of the cases by linkage with the National Services on Causes of Deaths and using information on causes of death [28]. Overall, 93% of the incident breast cancer cases were validated by pathology reports. Cases were included even when pathology reports were not available because of the very low percentage of self-reported false positives (<5%). Tumour characteristics, including histological type and hormone receptor status, were extracted from the pathology reports. When more than one breast tumour was diagnosed at the same time, we recorded the tumour-node-metastasis (TNM) stage for the most advanced breast tumour, or grade of differentiation, if the TNM stages were the same. A total of 6298 histologically confirmed incident invasive breast cancer cases were identified in the E3N-Generation cohort during the 1990–2011 follow-up period.

Each case was matched to a control, randomly selected in the E3N-Generation cohort by incidence density sampling, using at time since entry into the cohort as the time scale. Controls with a blood sample were matched on age ( ± 1 year), French Department of residence (INSEE, 2023), corresponding to “NUTS-3” in the classification of territorial divisions of the European Union (Eurostat, 2021), date of blood collection ( ± 3 months) and menopausal status at the time of blood collection. Controls without a blood sample were matched on the same criteria but these criteria were assessed at baseline (i.e., the time of entry into the cohort) rather than at blood collection, and were additionally matched on the availability or not of a saliva sample. Breast cancer cases were selected based solely on clinical criteria. The availability of a blood sample was not used for case selection but was applied as a matching criterion for selecting controls to enable future analyses.

Among the 6298 incident cases of primary invasive breast cancer and their 6298 matched controls initially involved in the study, women with Paget’s disease and phyllodes tumours (*n* = 19 cases, and their matched controls, 0.3%), those with missing matching variables (*n* = 3 women, and their matched women, 0.05%) and those with at least two missing addresses, as well those living abroad during follow-up time (*n* = 1054 women, and their matched women, 16.7%) were excluded [26, 28]. Thus, 5222 incident cases of primary invasive breast cancers and 5222 matched controls were included in this study.

### Assessment of long-term exposure to airborne PM_2.5_ and PM_10_

#### Estimation of atmospheric PM concentrations

The average annual atmospheric PM concentrations were estimated at the individuals’ residential areas each year from 1990 to 2011, using addresses collected through the E3N-Generation follow-up questionnaires. Two different models of exposure assessment were used to estimate PM concentrations: a land use regression (LUR) model, with a spatial resolution of 50 × 50 m, and an atmospheric chemistry-transport model named CHIMERE, (0.125° × 0.625° resolution, roughly 7 × 7 km) [28, 29]. LUR is a common statistical approach that models the spatial variability of air pollutants [30, 31]. The model is built up of several variables, mainly geographical features (e.g., land use, road networks, traffic or terrain) calculated from circular buffers of different sizes, considered to have an impact on local concentrations [30, 32, 33]. Baseline LUR model were first develop for 2010–2012, and validated against measurements across France (*n* = 94 and 336 for PM_2.5_ and PM_10_, respectively) by performing a hold-out validation. The monitoring sites were randomly split into five groups for each pollutant and we undertook 5-fold cross-validation by leaving one group out at a time, maintaining the same variables and allowing the coefficients to vary, to predict concentration values at sites in the held-out group. The summarized performance shows robust results with R² values of 0.56 for PM_2.5_ and 0.66 for PM_10_. Models were back-extrapolated to 1990, after assessment and comparison of four different back-extrapolation approaches against measurements. Model performance remains stable until 2005, after which it declines and fluctuates markedly. This pattern can partially be explained by the substantial reduction in the number of monitoring stations available, particularly before 2002. This limitation constrains the conclusions that can be drawn regarding past model performance but comparisons with baseline models indicate that back-extrapolation reduces the mean error by approximately 20%. These exposure models were already used in two epidemiological studies on breast cancer [34, 35]. The CHIMERE model, develop by the National Institute for Industrial Environment and Risks [36, 37], is the European reference chemistry transport model. The model use emission data, meteorological fields, and boundary conditions as inputs and computes a set of equations representing the physical and chemical processes involved in the evolution of concentrations. The performances of this model are detailed for 2013 at the European level by Couvidat et al. in comparison with daily concentrations (PM_10_: correlation: 0.6 / RMSE: 9.3; PM_2.5_ correlation: 0.68 / RMSE: 6.95) [38]. Data from the CHIMERE model has been used in several previous analyses in the same population investigating the effect of other pollutants on the risk of breast cancer [26, 35, 39, 40].CHIMERE is a Eulerian deterministic model that simulates pollutant atmospheric dispersion and other physical and chemical processes using emission data, meteorological fields, and boundary conditions as inputs, and provides hourly averaged concentrations from 1990 to 2010. As PM_2.5_ and PM_10_ concentrations were not available for 2011, concentrations were extrapolated using average change rates of the last 5 years available (2005 to 2010) for each address.

Since concentrations of PM_2.5_ and PM_10_ can have locally very high concentrations, near major roads for example [30, 32, 33], the main analyses of the study used exposure estimated by the LUR model (higher spatial resolution than CHIMERE). Sensitivity analyses for exposure to PM_2.5_ and PM_10_ were performed using the CHIMERE model [41].

The residential history of the participants was geocoded by trained technicians, blinded to the subjects’ case or control status, using the ArcGIS Software (ArcGIS Locator version 10.0, Environmental System Research Institute (ESRI), Redlands, CA, USA) and the address database, BD Address®, from the National Geographic Institute [29]. First, addresses were geocoded automatically and then 16.9% were manually relocated to improve their accuracy. Manual relocation was performed for addresses with low spatial precision or low textual accuracy, following previously described criteria [29]. If addresses were missing at any time during follow-up, the previously recorded address was systematically assigned [26].

Annual mean PM concentration values (in µg/m^3^) were assigned to the geocoded consecutive residential addresses of each woman, for each year, from inclusion in the cohort to the index date. If a woman moved within a year, the PM_2.5_ and PM_10_ concentrations were weighted by the time spent at each address. An average of the annual mean PM_2.5_ and PM_10_ concentration estimates was then calculated for each woman, by adding the concentrations estimates of each year from inclusion in the cohort to the index date and dividing by the number of years of follow-up.

### Statistical analyses

The statistical analyses for PM_2.5_ and PM_10_ were run separately, as they are considered as two different pollutants. Atmospheric exposure estimates for PM_2.5_ and PM_10_, socio-demographic characteristics and other covariates were described for cases and controls, using mean and standard deviation (SD) for continuous variables, and frequency and percentage for categorical variables. All characteristics were collected at baseline (1990), except for alcohol consumption, which was collected at the third questionnaire (1993). Information available in the questionnaire before the index date was used to determine menopausal status and menopausal hormonal replacement treatments. The distributions and evolution over time (1990–2011) of the mean estimated annual PM_2.5_ and PM_10_ concentrations at the different addresses of the participants were graphically described.

OR and corresponding 95% CIs for invasive breast cancer were estimated using conditional logistic regression models, with PM_2.5_ and PM_10_ exposure as continuous variables, for an increment of 10 µg/m^3^ to be homogenous with other studies. A directed acyclic graph (DAG) was used to select the sufficient set of minimal adjustment variables (Fig. S1). This resulted into two sets of variables. The first one included level of education (secondary, 1- or 2-year university degree, and ≥3-year university degree, used as a proxy for socio-economic status) and urban / rural location at inclusion. The second one involved total physical activity (<25.3, 25.3–35.5, 35.6–51.8, and ≥51.8, in metabolic equivalent task per hour per week (MET-h/week)), tobacco smoking status (never, current, former), alcohol drinking (never, ≤6.7 g/day, and >6.7 g/day), body mass index (BMI) (<25, 25–30, and ≥30 kg/m²), parity and age at first full-term pregnancy (no child, 1 or 2 children and age <30 years, 1 or 2 children and age ≥30 years, ≥3 children), breastfeeding (ever, never), oral contraceptive use (ever, never), menopausal hormone replacement therapy use (HRT) (ever, never), mammography before inclusion (yes, no), urban/rural location at inclusion, and urban/rural location at birthplace (urban and rural). We also considered a third set which included the first set and other variables identified as known and potential risk factors of breast cancer in the literature: age at menarche (<12, 12–14, and ≥14 years), menopausal status at index date (premenopausal, postmenopausal), previous family history of breast cancer (yes, no), and previous history of benign breast disease (yes, no). Subgroup analyses were also carried out using breast tumour hormone receptors status (oestrogen receptors (ER) and progesterone receptors (PR)) stage (I, II, III and IV), grade (I, II, III and IV) and histology subtypes of the tumour (ductal, lobular, tubular and, both ductal and lobular).

Assuming data were missing at random, multiple imputation was conducted for the following variables: alcohol intake (28.9% of missing data), urban/rural status of the municipality at birthplace (11.1% of missing data), age at menarche (2.0% of missing data), BMI (1.9% of missing data), oral contraceptive use (0.9% of missing data), family history of breast cancer (1.6% of missing data), parity and age at first full-term pregnancy (1.1% of missing data), education level (0.7% of missing data), smoking status (0.3% of missing data) and total physical activity (0.1% of missing data). We carried out 10 imputations, each with 10 iterations with a multivariate imputation via a chained equations approach (MICE) [42]. All variables included in the different adjustment models, matching variables and exposure variable were considered as predictors. Analyses were conducted in parallel across the 10 imputed datasets and subsequently pooled using Rubin’s rules.

Simple imputation was used for menopausal status at inclusion. If age at menopause was missing and a woman’s age at inclusion was less than 51 years (median age at menopause of women in the E3N cohort), women were considered premenopausal at inclusion, otherwise, women were considered postmenopausal at inclusion [43].

For women with missing data on oral contraceptive use at index date and having reported ‘never use’ at baseline, we assumed for these women that they continued ‘never use’ during follow-up, since age at cohort inclusion was 40 to 65 years. No imputation was performed for menopausal hormone therapy (MHT) as this variable is highly dependent on age and menopausal status, but a missing data category was created.

The linearity of the logit of the effect of the quantitative variables (PM_2.5_ and PM_10_ exposure) was investigated using second-order fractional polynomials.

The effect modification by total physical activity, BMI, level of education, tobacco smoking status, breastfeeding and menopausal status during follow-up were tested by including interaction terms between these variables and exposure to PM_2.5_ and PM_10_, using the first adjusted model. Menopausal status over the study period was analysed in three categories: premenopausal (women who self-reported premenopausal on the last returned questionnaire or an age at menopause greater than their age at the index date), postmenopausal (women who self-reported postmenopausal on the last returned questionnaire before the index date or an age at menopause younger than their age at the index date), and women premenopausal at study inclusion who transitioned to postmenopausal status before the index date (i.e., women whose status changed during follow-up). Premenopausal and postmenopausal status refer to women who remained in the same category throughout the follow-up period. The third category was defined based on evidence that the menopausal transition represents a window of susceptibility to environmental exposures, particularly those with endocrine-disrupting properties, due to substantial hormonal and structural changes in breast tissue during this period [44–46].

A sensitivity analysis was performed using only matched pairs that had been followed for at least 2 years to analyse only data for women with a relatively long follow-up time since breast cancer is known to have a long period of latency.

All statistical analyses were performed with SAS version 9.4 and R software version 4.2.0 and the threshold for statistical significance was set at 5%.

## Results

### Characteristics of study population

The characteristics of the study population, which have been previously described, are summarised in Table S1 [26]. Briefly, cases were more likely to be older than 30 at first birth (13.0% vs. 10.7%), have less than 3 children (73.4% vs. 69.7%), have had a mammography before inclusion (77.1% vs. 72.7%), have a family history of breast cancer (17.0% vs. 10.6%), and have a previous history of benign breast disease (29.4% vs. 22.6%). The average annual concentration of atmospheric exposure, estimated by the LUR models, was 26.4 µg/m³ ( ± 6.2) for PM_2.5_ and 35.8 µg/m³ ( ± 8.4) for PM_10_ among cases, while it was 26.3 µg/m³ ( ± 6.1) for PM_2.5_ and 35.6 µg/m³ ( ± 8.2) for PM_10_ among controls.

**Fig. 1:**
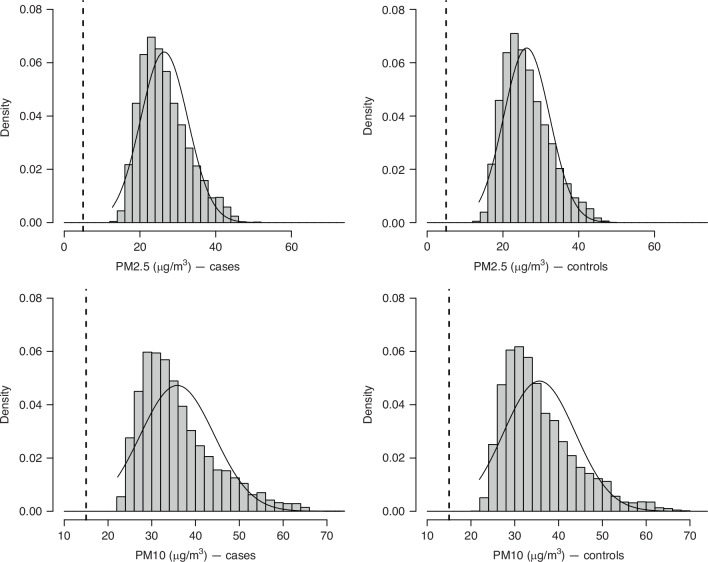
Distribution of annual average PM_2.5_ and PM_10_ concentration estimates for 5222 cases and 5222 controls during follow-up (from inclusion to index date), using a LUR model. XENAIR case–control study nested in the E3N-Generation cohort, France, 1990–2011. Distribution of PM_2.5_ concentration estimates and the corresponding 2021 WHO reference threshold (vertical dashed lines) for cases on the top left and for controls on the top right. Distribution of PM_10_ concentration estimates with the corresponding 2021 WHO reference threshold (vertical dashed lines) for cases on the bottom left and for controls on the bottom right.

**Fig. 2:**
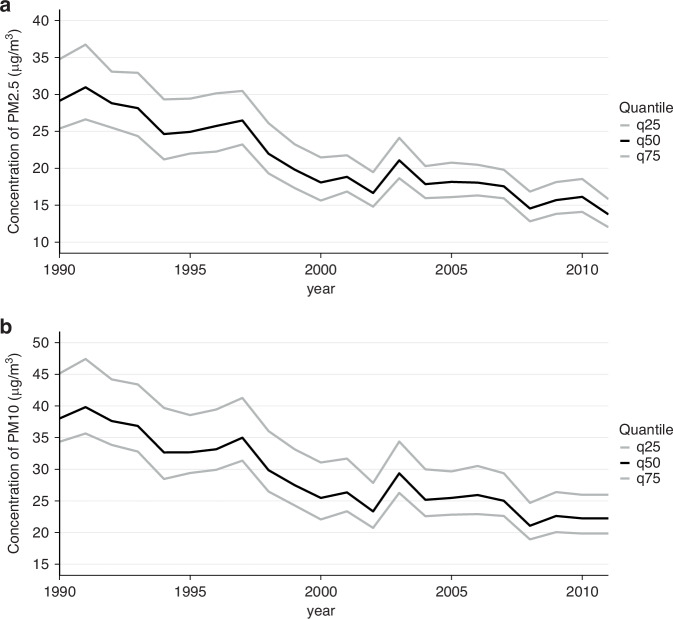
Evolution (median, 1st and 3rd quartiles) of the mean annual land use regression (LUR) concentration estimates at the residential addresses of the study participants from 1990 to 2011, for PM_2.5_ and PM_10_ concentrations. **a** PM_2.5_ and **b** PM_10_ concentrations. XENAIR case–control study nested in the E3N-Generation cohort, France, 1990–2011.

The distributions of the average PM_2.5_ and PM_10_ concentrations between inclusion and index date in cases and controls, estimated by the LUR models, were almost normal and were centred around approximately 25 µg/m^3^ and 35 µg/m^3^, respectively, with a similar distribution for cases and controls (Fig. 1). In 1990, the median of the mean annual PM_2.5_ and PM_10_ concentrations levels at the addresses of the participating women were 29.1 µg/m^3^ (interquartile range (IQR): 9.3 µg/m^3^) and 38.0 µg/m^3^ (IQR: 10.5 µg/m^3^), respectively (Fig. 2). The concentrations of both PM_2.5_ and PM_10_ decreased rapidly until 2000 and then the decrease was slower between 2000 and 2011, with a peak in 2003, corresponding to a major heatwave that year. In 2011, the median of the mean annual PM_2.5_ and PM_10_ concentrations of PM_2.5_ and PM_10_ at residential addresses were 16.2 µg/m^3^ (IQR: 4.3 µg/m^3^) and 22.2 µg/m^3^ (IQR: 5.7 µg/m^3^).

### Mean exposure to PM_2.5_ and PM_10_ and breast cancer risk

For exposure to PM_2.5_ and PM_10_, estimated using LUR models, the results for model I (adjusted for level of education and urban/rural status at inclusion) were, for an increase of 10 µg/m^3^ OR = 1.14 (95% CI: 0.99–1.30) and OR = 1.08 (95% CI: 0.98–1.18), respectively. The ORs for the association of the mean exposure to PM_2.5_ and PM_10_ were similar across the three different adjustment models (Table 1).

**Table 1:** Adjusted odds ratios (ORs) and 95% confidence intervals (CIs) for the association between invasive breast cancer and mean exposure to PM, estimated with the LUR model, among 5222 cases and 5222 controls, XENAIR case-control study nested in the E3N-Generation cohort, France, 1990–2011.

	Model I^a^	Model II^b^	Model III^c^
	OR (95% CI)	OR (95% CI)	OR (95% CI)
PM_2.5_ mean exposure	1.14 (0.99–1.30)	1.12 (0.97–1.29)	1.13 (0.98–1.30)
PM_10_ mean exposure	1.08 (0.98–1.18)	1.06 (0.97–1.16)	1.07 (0.97–1.17)

PM_2.5_, particulate matters with a diameter <2.5 µm; PM_10_, particulate matters with a diameter <10 µm.

The OR (95% CI) corresponds to an increment of 10 µg/m^3^ of the average of the mean annual concentrations.

^a^Adjusted for level of education and urban/rural status at inclusion.

^b^Adjusted for urban/rural status at inclusion, urban/rural status at birthplace, total physical activity, smoking status, alcohol drinking, body mass index, age at first full-term pregnancy, parity, breastfeeding, oral contraceptive use, menopausal hormone replacement therapy use and mammography before inclusion.

^c^Adjusted for the model I variables and also for age at menarche, menopausal status at index date, previous family history of breast cancer and personal history of benign breast disease.

When stratifying by menopausal status during follow-up, no statistically significant associations were observed; moreover, no effect modification by menopausal status was detected (*p* = 0.99 for PM_2.5_ and 0.93 for PM_10_) (Table 2). The results of the association of PM_2.5_ and PM_10_ exposure (estimated with the LUR model) and breast cancer, grade, stage and histological type subgroups, and by hormone receptor status (known for 72% of the women) are summarised in Table 3. By histology subtypes, the ORs for the association between mean exposure to PM_2.5_ and PM_10_ and breast cancer remained statistically significant only for invasive ductal and lobular carcinomas were 2.74 (95% CI: 1.05–7.15) and 2.05 (95% CI: 1.11–3.78), respectively. For stage I breast cancer, the ORs for PM_2.5_ and PM_10_ were 1.24 (95% CI: 1.03–1.49) and 1.15 (95% CI: 1.02–1.30), respectively. The 95% CIs for the ORs for the other subgroups were not statistically significant (Table 3). No association was found by hormonal status (Table 3). There was no evidence of effect modification by level of education, physical activity, BMI, tobacco smoking status and breastfeeding (*P* for interaction >0.05, Table S4).

**Table 2:** Adjusted odds ratios (ORs) and 95% confidence intervals (CIs) for the association between invasive breast cancer and mean exposure to PM^a^, by menopausal status during follow-up.

		PM_2.5_ mean exposure		PM_10_ mean exposure	
	Matched pairs (1:1)	Adjusted OR^b^ (95% CI)	*P*-value^c^	Adjusted OR^b^ (95% CI)	*P*-value^c^
Premenopausal	591	1.11 (0.79–1.56)	0.99	1.01 (0.81–1.27)	0.93
Change in menopausal status^d^	1958	1.07 (0.85–1.35)		1.03 (0.88–1.20)	
Postmenopausal	1991	1.11 (0.90–1.38)		1.06 (0.93–1.22)	

XENAIR case-control study nested in the E3N-Generation cohort, France, 1990–2011.

PM_2.5_, particulate matters with a diameter <2.5 µm; PM_10_, particulate matters with a diameter <10 µm.

Matched pairs (1:1): number of case-control pairs included in the analyses. Each pair consists of one case and one matched control.

The OR (95% CI) corresponds to an increment of 10 µg/m^3^ of mean exposure.

^a^Exposure to PM was estimated using a LUR model.

^b^Adjusted for level of education and urban/rural status at inclusion.

^c^*p*-values derived from the likelihood ratio test testing the interaction terms.

^d^Women who were premenopausal at inclusion and were postmenopausal at index date.

**Table 3:** Adjusted odds ratios (ORs) and 95% confidence intervals (CIs) for the association between invasive breast cancer and mean exposure to PM_2.5_ and PM_10_^a^ by grade of differentiation, stage, histology subtype and hormone receptor status of the tumor.

		PM_2.5_ mean exposure	PM_10_ mean exposure
	Matched cases/controls^b^	Adjusted OR^c^ (95% CI)	Adjusted OR^c^ (95% CI)
Grade			
Grade 1	614/614	1.24 (0.84–1.83)	1.14 (0.89–1.48)
Grade 2	1483/1483	1.01 (0.78–1.30)	0.98 (0.84–1.16)
Grade 3	2067/2067	1.09 (0.87–1.35)	1.09 (0.94–1.26)
Stage			
Stage I	2919/2919	1.24 (1.03–1.49)	1.15 (1.02–1.30)
Stage II	1412/1412	1.10 (0.85–1.42)	1.05 (0.89–1.25)
Stage III and Stage IV^d^	402/402	0.76 (0.47–1.22)	0.82 (0.60–1.11)
Histology subtypes			
Invasive ductal	3568/3568	1.15 (0.97–1.36)	1.09 (0.97–1.22)
Invasive lobular	828/828	0.92 (0.66-1.27)	0.91 (0.73–1.14)
Invasive tubular	141/141	1.07 (0.48–2.37)	1.15 (0.66–2.00)
Invasive ductal and lobular	123/123	2.74 (1.05–7.15)	2.05 (1.11–3.78)
Hormone receptor status			
ER+	3405/3405	1.17 (0.98–1.39)	1.08 (0.96–1.21)
ER-	760/760	1.04 (0.71–1.51)	1.06 (0.82–1.35)
PR+	2602/2602	1.15 (0.94–1.40)	1.06 (0.92–1.21)
PR-	1439/1439	1.07 (0.82–1.39)	1.04 (0.88–1.24)
ER + /PR+	2459/2459	1.13 (0.92–1.39)	1.04 (0.91–1.20)
ER-/PR-	612/612	0.90 (0.59–1.38)	0.97 (0.73–1.28)
ER + /PR-	825/825	1.18 (0.84–1.65)	1.08 (0.87–1.36)
ER-/PR+	140/140	1.48 (0.63–3.52)	1.32 (0.75–2.32)

XENAIR case-control study nested in the E3N-Generation cohort, France, 1990–2011.

OR: Odds ratio; 95% CI: 95% confidence intervals; PM_2.5_: particulate matters with a diameter <2.5 µm; PM_10_: particulate matters with a diameter <10 µm

ORs (95% CI) correspond to a mean exposure increment of 10 µg/m^3^.

Data were missing for grade (*n* = 1058), stage (*n* = 489) and histology subtype (*n* = 281).

^a^Exposure to PM was estimated using a LUR model.

^b^The sum does not add up to the total because the matching pairs were maintained.

^c^Adjusted for level of education and urban/rural status at inclusion.

^d^Stage III and stage IV were considered together because only one woman had stage IV cancer.

As sensitivity analyses, the adjusted ORs for the associations of mean PM_2.5_ and PM_10_ exposure, and risk of breast cancer, after exclusion of those who were diagnosed with cancer after less than two years of follow-up, were 1.12 (95% CI: 0.97–1.30) and 1.07 (95% CI: 0.98–1.18), respectively (Table S3).

When the mean PM_2.5_ and PM_10_ exposures were estimated with the CHIMERE model, the adjusted ORs, for a mean exposure increment of 10 µg/m^3^, were 1.28 (95% CI: 1.12–1.46) and 1.26 (95% CI: 1.11–1.42), respectively (Table S2).

## Discussion

To the best of our knowledge, this large nested case-control study is the first study to use two distinct models (LUR and CHIMERE) to estimate the association of PM_2.5_ and PM_10_ exposure with the risk of female breast cancer up to a 22-year period. The mean concentrations, particularly for PM_2.5_, were above the 2005 and 2021 thresholds of the WHO air quality guidelines [8]. Long-term exposure to PM_2.5_ and PM_10_ were both associated with an increased risk of breast cancer, with ORs of 1.14 (95% CI: 0.99–1.30) and 1.08 (95% CI: 0.98–1.18), respectively, per 10 µg/m^3^ increase in the average of PM_2.5_ and PM_10_ over the study period. By subgroups, long-term exposure to PM_2.5_ and PM_10_ appeared to be associated with an increased risk of mixed invasive ductal and lobular carcinomas and stage I breast cancers. The high association observed for women diagnosed with both invasive ductal and lobular carcinoma may reflect distinct tumour biology, possibly with increased hormone sensitivity, making such tumours more responsive to endocrine-disrupting exposures like PM [47]. However, given the limited number of cases in this subgroup, this result should be interpreted with caution. The association observed for stage I cancers may partly reflect the higher number of cases in this category, which provides greater statistical power. It may also indicate that PM exposure has a stronger influence on early carcinogenic events rather than on progression to later stages, although this requires further investigation [48].

Our results suggest a potential association between PM exposure and ER+ breast cancer. This could be explained by the presence, within PM, of endocrine-disrupting chemicals (e.g., PAHs, heavy metals), which may interfere with estrogen signalling [7, 48, 49]. These compounds can mimic or alter hormone activity, induce oxidative stress and inflammation, and promote epigenetic modifications in hormone-sensitive tissues, potentially contributing to ER+ tumour development [7, 49]. Such mechanisms support the plausibility of a differential effect of PM by hormone receptor status.

Consistent results were observed with both exposure assessment methods, i.e., the LUR and the CHIMERE methods. The consistency of the results is reinforced by the similarity of the associations obtained with the two models, even though they differ on several points. The LUR model is a statistical approach, which is calibrated and validated on measurements to obtain fine spatial resolution while the CHIMERE model is a deterministic model that solves fluid mechanics equations to simulate the behaviour of pollutants in the air. Due to the spatial resolution of the emissions data used (EMEP European emissions database), the spatial resolution of the concentrations estimated with CHIMERE is modest (7 × 7 km). This model will therefore tend to smooth out the very high concentrations present on fine spatial scales (close to roads, for example), and provide a more restricted set of exposures than the LUR model.

Evidence for an association between PM_2.5_ and PM_10_ and the risk of female breast cancer is conflicting [13, 17, 21, 50]. Our results are compatible with those from a population-based study in Denmark that used an integrated chemical transport air pollution model to estimate PM_2.5_ exposure at the residential addresses over 20 years prior to the index date [21]. They reported an OR of 1.32 (95% CI: 1.09–1.60) for PM_2.5_ exposure in the 20 years prior to breast cancer diagnosis in women under 55 years old, but the median exposure levels were lower than those reported in the present study. In another study in the US, long-term exposure to PM_2.5_ and PM_10_ was found to be associated with increased risk of both invasive and ductal carcinoma in situ breast cancer, but reported variations by region and age group [19, 51]. In this latter study, exposure estimates were based on the annual average concentrations at their residential addresses in the 12 months prior to enrolment.

Differences in the PM exposure assessment methods used, in terms of spatial resolution (from <100 metres to 10 kilometres), geographical coverage (regional to national coverage) and temporal resolution (i.e., data updated daily to data updated annually over a highly variable period which can extend over more than 10 years) could partly explained the conflicting conclusions [17, 23]. In one meta-analysis, the level of exposure was low, with an overall mean of 10.9 µg/m^3^ for PM_2.5_ and 20.9 µg/m^3^ for PM_10_ [17]. In addition, some studies only used addresses at inclusion or diagnosis to estimate exposure throughout the study periods. Given the long latency period for development of breast cancers, one of the main challenges is to have accurate long-term data for PM_2.5_ and PM_10_ exposure [52].

One of the strengths of this study is the large population size that provided relatively good statistical power, which is important as the reported measures of association between PM_2.5_ and PM_10_ and breast cancer are generally low. In addition, the prospective cohort design and the long-term follow-up enabled data for covariates to be reliably collected and, therefore, to control for confounding factors that could potentially impact the association. Another strength of our study is the use of two complementary exposure assessment methods, including one with a fine spatial resolution (50 × 50 m), and the CHIMERE model (7 × 7 km) to estimate the average annual exposure over 22 years and another, which provided consistent results. The annual evolution of PM_2.5_ and PM_10_ exposure was found to decrease relatively rapidly from 1990 to 2000 and then more gradually from 2000 to 2011. This approach enabled the participants’ exposure to PM_2.5_ and PM_10_ to be assessed in terms of their residential trajectories over the follow-up of up to 22 years, rather than just in terms of one residence location, at inclusion or at breast cancer diagnosis, as has often been done in other studies [17]. Moreover, the long-term follow-up also made it possible to measure the effects of chronic exposure to PM_2.5_ and PM_10_, whereas most other studies measure exposure over a shorter period [17].

One of the weaknesses of the study, as with many others, is the lack of PM_2.5_ and PM_10_ exposure data during specific windows of susceptibility such as childhood, puberty, menarche and pregnancy, which occurred prior to study entry since the women were enrolled between 45 and 60 years of age [53]. During these windows, significant structural and functional changes occur in the mammary gland, the mammary micro-environment is modified and hormone signalling occurs, all of which may affect female breast cancer risk. However, recent results from the National Institutes of Health—AARP Diet and Health Study showed a strong correlation between PM_2.5_ levels over time at a single location and breast cancer [51]. In addition, the reported estimates were similar across the three time periods assessed (1980–1984, 1985–1989, 1990–1994) [51].

In our study, the estimated PM_2.5_ and PM_10_ exposures did not take into account workplace exposure, exposure during commuting or indoor exposure. However, in a subset of the present population (2416 breast cancer cases and 2984 controls), we investigated the effect of PM_2.5_ and PM_10_ exposure estimated at both residential and workplace locations [34]. The findings from this analysis were of the same magnitude as those reported in the present study, suggesting that considering workplace exposure does not substantially change the overall results in this population. In addition, PM_2.5_ and PM_10_ are complex, heterogeneous mixtures, and the risk associated with the individual components, or the correlated risk associated with certain mixtures were not assessed [19]. The compositions of PM_2.5_ and PM_10_ were considered stable, whereas these may vary geographically and over time [19].

Additionally, information on detailed occupational exposure potentially related to breast cancer risk was not available. However, women in the E3N-Generation cohort were teachers or worked in affiliated occupations, thus their occupational exposure was relatively low and homogeneous. Also, despite the extensive efforts to adjust for potential confounding factors, we cannot exclude the possibility of residual confounders such as noise exposure or other pollutants. Confounding from dietary exposure was not expected since inhalation is the only route of exposure to PM in the general population that could impact health. The E3N-Generation cohort is composed of women in a national health insurance plan that mainly covers teachers at all levels (primary, secondary, and higher education). This implies they had a higher level of education than the general population of women which could result in healthier behaviours, although the women in the study would have been exposed to similar levels of PM as those in the general population (mean gap between the PM concentration of the municipalities of the XENAIR population and the PM concentration of municipalities of the French population in 1990–2011, is 0.01 µg/m^3^, corresponding 0.1% of relative change).

The results presented here suggest a potential association between PM_2.5_ and PM_10_ exposure and risk of female breast cancer, with the highest associated risk observed for mixed ductal and lobular breast cancers and higher risk observed for stage I breast cancer. However, further long-term exposure studies, with precise and consistent assessments of atmospheric exposure over time, are required to confirm this association.

## Supplementary information

Supplementary materials

## Data Availability

The datasets analysed during the current study may be available from the corresponding author under on reasonable request.
