# Supplementary material for: Long-term atmospheric exposure to particulate matter and breast cancer risk: findings from a nested case-control study in France
Source: Br J Cancer. 2026 Jan 13;134(7):1092–100. doi: 10.1038/s41416-025-03311-y (PMC12996323; doi:10.1038/s41416-025-03311-y)
Supplement: Supplementary file 1 — Supplementary materials [file 41416_2025_3311_MOESM1_ESM.docx]

**Supplementary materials**

**Table S1.** Characteristics of cases and controls in the XENAIR case-control study nested in the E3N-Generation cohort, France, 1990-2011.

| **Characteristics** | **Cases (n = 5,222)**  **mean** ± SD or **n (%)** | **Controls (n = 5,222)**  **mean ± SD or n (%)** |
| --- | --- | --- |
| Age at baseline (years) | 49.6 ± 6.3 | 49.5 ± 6.3 |
| Age at index date (years) | 60.6 ± 8.1 | 60.6 ± 8.1 |
| Time to index date (years) | 11.0 ± 5.7 | 11.1 ± 5.7 |
| LUR airborne PM_2.5_ (µg/m^3^) (min-max, IQR) | 26.4 ± 6.2  (12.7-50.2, 8.49) | 26.3 ± 6.1  (13.7-48.8, 8.29) |
| CHIMERE airborne PM_2.5_ (µg/m^3^) (min-max, IQR) | 19.1 ± 6.1  (6.7-41.9, 7.3) | 18.8 ± 5.8  (6.8-41.3, 7.1) |
| LUR airborne PM_10_ (µg/m^3^) (min-max, IQR) | 35.8 ± 8.4  (22.2-73.1, 10.62) | 35.6 ± 8.2  (21.9-69.3, 10.31) |
| CHIMERE airborne PM_10_ (µg/m^3^) (min-max, IQR) | 21.6 ± 6.3  (7.8-45.1, 7.5) | 21.4 ± 6.0  (7.9-44.5, 7.2) |
| Body mass index (kg/m^2^) at baseline |  |  |
| < 25 | 4,236 (81.1) | 4,239 (81.2) |
| 25-29 | 744 (14.2) | 715 (13.7) |
| ≥ 30 | 149 (2.9) | 162 (3.1) |
| Missing | 93 (1.8) | 106 (2.0) |
| Alcohol consumption at questionnaire 3 |  |  |
| Never (n, %) | 402 (7.7) | 473 (9.1) |
| ≤ 6.7 (g/day) | 1,291 (24.7) | 1,391 (26.6) |
| > 6.7 (g/day) | 2,002 (38.3) | 1,865 (35.7) |
| Missing (n, %) | 1,527 (29.2) | 1,493 (28.6) |
| Smoking status at baseline |  |  |
| Never smoker | 2,800 (53.6) | 2,861 (54.8) |
| Current smoker | 781 (15.0) | 743 (14.2) |
| Former smoker | 1,626 (31.1) | 1,603 (30.7) |
| Missing | 15 (0.3) | 15 (0.3) |
| Urban/rural status of the birthplace municipality |  |  |
| Rural | 1,369 (26.2) | 1,429 (27.4) |
| Urban | 3,299 (63.2) | 3,190 (61.1) |
| Missing | 554 (10.6) | 603 (11.5) |
| Urban/rural status at the baseline municipality |  |  |
| Rural | 1,558 (29.8) | 1,592 (30.5) |
| Urban | 3,664 (70.2) | 3,630 (69.5) |
| Total physical activity (METs-h/week) at baseline |  |  |
| < 25.3 | 1,304 (25.0) | 1,229 (23.5) |
| 25.3-35.5 | 1,388 (26.6) | 1,359 (26.0) |
| 35.6-51.8 | 1,341 (25.7) | 1,322 (25.3) |
| ≥ 51.8 | 1,187 (22.7) | 1,299 (24.9) |
| Missing | 2 (0.0) | 13 (0.2) |
| Education |  |  |
| Secondary | 802 (15.4) | 874 (16.7) |
| 1- to 2-year university degree | 2,460 (47.1) | 2,584 (49.5) |
| ≥ 3-year university degree | 1,923 (36.8) | 1,727 (33.1) |
| Missing | 37 (0.7) | 37 (0.7) |
| Age at menarche (years) |  |  |
| <12 | 1,098 (21.0) | 1,050 (20.1) |
| 12-13 | 2,604 (49.9) | 2,588 (49.6) |
| ≥ 14 | 1,420 (27.2) | 1,470 (28.2) |
| Missing | 100 (1.9) | 114 (2.2) |
| Previous use of oral contraceptives at baseline |  |  |
| Yes | 3,075 (58.9) | 3,064 (58.7) |
| No | 2,086 (39.9) | 2,127 (40.7) |
| Missing | 61 (1.2) | 31 (0.6) |
| Menopausal status at baseline |  |  |
| Premenopausal | 3107 (59.5) | 3127 (59.9) |
| Postmenopausal | 2072 (39.7) | 2069 (39.6) |
| Missing | 43 (0.8) | 26 (0.5) |
| Menopausal status at index date |  |  |
| Premenopausal | 873 (16.7) | 804 (15.4) |
| Postmenopausal | 4,306 (82.5) | 4,392 (84.1) |
| Missing | 43 (0.8) | 26 (0.5) |
| Use of hormone replacement therapy |  |  |
| Yes | 3,064 (58.7) | 2,913 (55.8) |
| No | 2,029 (38.9) | 2,187 (41.9) |
| Missing | 129 (2.5) | 122 (2.3) |
| Mammography during the previous follow-up period |  |  |
| Yes | 4,026 (77.1) | 3,795 (72.7) |
| No | 1,196 (22.9) | 1,427 (27.3) |
| Parity |  |  |
| 0 | 674 (12.9) | 562 (10.8) |
| 1-2 | 3,163 (60.5) | 3,075 (58.9) |
| ≥ 3 | 1,350 (25.9) | 1,559 (29.9) |
| Missing | 35 (0.7) | 26 (0.5) |
| Age at first full-term pregnancy (years) |  |  |
| Never pregnant | 674 (12.9) | 562 (10.8) |
| < 30 | 3,795 (72.7) | 4,034 (77.3) |
| ≥ 30 | 681 (13.0) | 558 (10.7) |
| Missing | 72 (1.4) | 68 (1.3) |
| Breastfeeding |  |  |
| Yes | 2,755 (52.8) | 2,786 (53.4) |
| No | 2,383 (45.6) | 2,373 (45.4) |
| Missing | 84 (1.6) | 63 (1.2) |
| Family history of breast cancer at baseline |  |  |
| Yes | 886 (17.0) | 555 (10.6) |
| No | 4,248 (81.3) | 4,584 (87.8) |
| Missing | 88 (1.7) | 83 (1.6) |
| Personal history of benign breast disease |  |  |
| Yes | 1,534 (29.4) | 1,177 (22.6) |
| No | 3,688 (70.6) | 4,045 (77.4) |

SD, standard deviation; MET, metabolic equivalent of task

All variables were considered at inclusion through the first questionnaire (Q1), except for urban/rural status which was considered at birth, alcohol drinking which was considered from the dietary questionnaire (Q3), for hormonal treatments (oral contraceptives and hormone replacement therapy), the information available in the last questionnaire before the index date was used and menopausal status was considered both at inclusion and index date.

**Table S2**. Adjusted odds ratios (ORs) and 95% confidence intervals (CIs) for the association between invasive breast cancer and mean exposure to PM_2.5_ and PM_10_, estimated with a CHIMERE model. XENAIR case-control study nested in the E3N-Generation cohort, France, 1990-2011.

|  | Matched  cases/controls  N/N | Adjusted OR^a^ (95% CI) |
| --- | --- | --- |
| PM_2.5_ mean exposure | 5222/5222 | 1.28 (1.12-1.46) |
| PM_10_ mean exposure | 5222/5222 | 1.26 (1.11-1.42) |

PM_2.5_: particulate matters with a diameter < 2.5 µg/m^3^;

PM_10_: particulate matters with a diameter < 10 µg/m^3^

The OR (95% CI) corresponds to an increment of 10 µg/m^3^ of mean exposure

^a^ Adjusted for level of education and urban/rural status at inclusion

**Table S3.** Adjusted odds ratios (ORs) and 95% confidence intervals (CIs) for the association between invasive breast cancer and mean exposure to PM_2.5_ and PM_10_ (estimated with LUR model), after excluding cases diagnosed during the first 2 years of follow-up. XENAIR case-control study nested in the E3N-Generation cohort, France, 1990-2011.

|  | Matched cases/controls  N/N | Adjusted OR^a^ (95% CI) |
| --- | --- | --- |
| PM_2.5_ mean exposure | 4864/4864 | 1.12 (0.97-1.30) |
| PM_10_ mean exposure | 4864/4864 | 1.07 (0.98-1.18) |

OR: Odds ratio; 95% CI: 95% confidence intervals;

PM_2.5_: particulate matters with a diameter < 2.5 µg/m^3^; PM_10_: particulate matters with a diameter < 10 µg/m^3^

ORs (95% CI) correspond to an increment of 10 µg/m^3^ of mean exposure

^a^ Adjusted for level of education and urban/rural status at inclusion

**Table S4.** Adjusted odds ratios (ORs) and 95% confidence intervals (95% CIs) for the association between invasive breast cancer and mean exposure to PM estimated with the LUR model. Effect modification analyses, XENAIR case-control study nested in the E3N-Generation cohort, France, 1990-2011.

|  |  | **PM_2.5_ (for an increment of 10µg/m^3^)** | | **PM_10_ (for an increment of 10µg/m^3^)** | |
| --- | --- | --- | --- | --- | --- |
|  | Cases/controls | OR^a^ (95% CI) | p^b^ | OR^a^ (95% CI) | p^b^ |
| Education level |  |  | 0.88 |  | 0.58 |
| Secondary | 145/145 | 0.81 (0.31-2.07) |  | 0.71 (0.36-1.40) |  |
| 1- to 2-year university degree | 1,203/1,203 | 0.96 (0.71-1.30) |  | 0.99 (0.80-1.23) |  |
| ≥ 3-year university degree | 700/700 | 1.04 (0.76-1.41) |  | 1.03 (0.85-1.24) |  |
| Physical activity (METs-h/week) |  |  | 0.94 |  | 0.79 |
| < 25.3 | 365/365 | 0.98 (0.64-1.49) |  | 0.94 (0.72-1.23) |  |
| 25.3-35.5 | 355/355 | 0.85 (0.53-1.38) |  | 0.88 (0.63-1.21) |  |
| 35.6-51.8 | 334/334 | 1.07 (0.60-1.90) |  | 1.01 (0.71-1.45) |  |
| ≥ 51.8 | 314/314 | 0.87 (0.46-1.64) |  | 0.75 (0.47-1.19) |  |
| Body mass index (kg/m^2^) |  |  | 0.71 |  | 0.45 |
| < 25 | 3,471/3,471 | 1.18 (1.01-1.41) |  | 1.14 (1.02-1.27) |  |
| ≥ 25 | 186/186 | 1.01 (0.53-1.95) |  | 0.95 (0.62-1.46) |  |
| Tobacco smoking-status |  |  | 0.83 |  | 0.74 |
| Never smoker | 1,575/1,575 | 1.08 (0.83-1.40) |  | 1.03 (0.87-1.23) |  |
| Current smoker | 126/126 | 1.25 (0.64-2.47) |  | 1.22 (0.81-1.86) |  |
| Former smoker | 532/532 | 1.21 (0.80-1.84) |  | 1.09 (0.82-1.43) |  |
| Breastfeeding |  |  | 0.99 |  | 0.57 |
| Yes | 1,496/1,496 | 1.16 (0.89-1.50) |  | 1.04 (0.87-1.24) |  |
| No | 1,118/1,118 | 1.18 (0.90-1.55) |  | 1.11 (0.93-1.32) |  |

PM_2.5_, particulate matters with a diameter < 2.5 µg/m^3^; PM_10_, particulate matters with a diameter < 10 µg/m^3^

The OR (95% CI) corresponds to an increment of 10 µg/m^3^ of mean exposure

^a^Adjusted for level of education and urban/rural status at inclusion

^b^ P-values derived from a likelihood ratio test


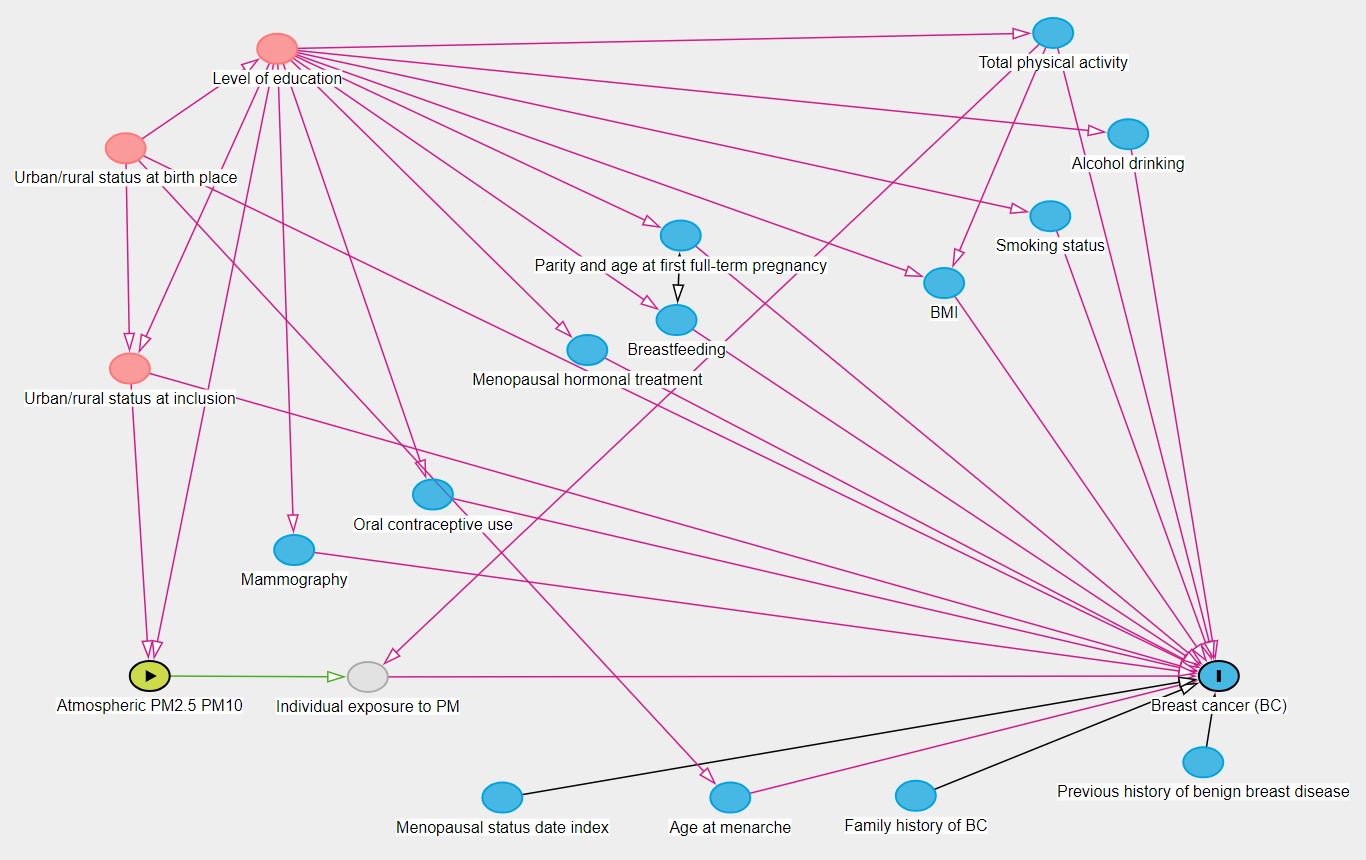


*BMI: body mass index*

**Figure S1 Directed acyclic graph (DAG) of the association between atmospheric exposure to PM_2.5_ and PM_10_ and breast cancer**
